# Molecular Reconstruction of an Old Pedigree of Diploid and Triploid *Hydrangea macrophylla* Genotypes

**DOI:** 10.3389/fpls.2018.00429

**Published:** 2018-04-18

**Authors:** Peter Hempel, Annette Hohe, Conny Tränkner

**Affiliations:** ^1^Leibniz Institute of Vegetable and Ornamental Crops, Erfurt, Germany; ^2^Faculty of Landscape Architecture, Horticulture and Forestry, University of Applied Sciences Erfurt, Erfurt, Germany

**Keywords:** genotype identification, gene bank, interploid crosses, microsatellite, SSR, fingerprint, ornamental crop

## Abstract

The ornamental crop species *Hydrangea macrophylla* exhibits diploid and triploid levels of ploidy and develops lacecap (wild type) or mophead inflorescences. In order to characterize a *H. macrophylla* germplasm collection, we determined the inflorescence type and the 2C DNA content of 120 plants representing 43 cultivars. We identified 78 putative diploid and 39 putative triploid plants by flow cytometry. In our collection 69 out of 98 flowering plants produced lacecap inflorescences, whereas 29 plants developed mophead inflorescences. Surprisingly, 12 cultivars included diploid as well as triploid plants, while 5 cultivars contained plants with different inflorescence types. We genotyped this germplasm collection using 12 SSR markers that detected 2–7 alleles per marker, and identified 51 different alleles in this collection. We detected 62 distinct fingerprints, revealing a higher genetic variation than the number of cultivars suggested. Only one genotype per cultivar is expected due to the vegetative propagation of *Hydrangea* cultivars; however we identified 25 cultivars containing 2–4 different genotypes. These different genotypes explained the variation in DNA content and inflorescence type. Diploid and triploid plants with the same cultivar name were exclusively mix-ups. We therefor assume, that 36% of the tested plants were mislabeled. Based on the “Wädenswil” pedigree, which includes 31 of the tested cultivars, we predicted cultivar-specific fingerprints and identified at least 21 out of 31 cultivars by SSR marker-based reconstruction of the “Wädenswil” pedigree. Furthermore, we detected 4 putative interploid crosses between diploid and triploid plants in this pedigree. These interploid crosses resulted in diploid or/and triploid offspring, suggesting that crosses with triploids were successfully applied in breeding of *H. macrophylla*.

## Introduction

*Hydrangea macrophylla* (thunb.) ser. is an economically important crop of the upmarket segment of ornamentals. *H. macrophylla* cultivars are famous for their rich foliage and impressively large, colorful inflorescences. Inflorescences can be divided into the lacecap (wild type) and the mophead (hortensia) type, according to the position and number of decorative flowers in the inflorescence. The lacecap inflorescence develops mainly non-decorative flowers, while several decorative flowers are present at the margin of the inflorescence. In contrast, the mophead inflorescence develops more decorative flowers, which are distributed over the complete inflorescence (Uemachi and Okumura, 2012). *H. macrophylla* originates from Japan and has been bred to create attractive ornamental pot plants and for landscaping in temperate regions worldwide. As for many ornamental crops, *Hydrangea* plants are commercially propagated through clones via cuttings. Mostly, the method of clone breeding is applied, where new varieties are selected in the F_1_ generation, followed by vegetative propagation. Thus, most cultivars possess a distinctive, predominantly heterozygous genotype. In addition, some varieties were obtained by mutant selection (Guérin, 2002). The fluctuation of established *Hydrangea* varieties is slow. For instance, varieties like ‘Mariesii’ (1879), ‘Madame Emile Mouillère’ (1909), ‘Libelle’ (1964) or ‘Blaumeise’ (1979) are still on the market.

Several famous lacecap cultivars such as ‘Libelle’ and ‘Blaumeise’ were derived from a systematic breeding program performed at the Experimental Station for Fruit Production, Viticulture and Horticulture in Wädenswil, Switzerland (nowadays Agroscope Changins-Wädenswil Research Station, ACW). This program had started in 1952 by crossing the mophead cultivar ‘Tödi’ with a lacecap wild type. It ended in 1987 after the release of 26 lacecap cultivars. In 1990, the complete pedigree of these lacecap cultivars was published (Meier, 1990). This pedigree, subsequently named “Wädenswil”, is a unique documentation of 36 years of systematic *Hydrangea* breeding. Noteworthy, all “Wädenswil” lacecap cultivars are based on one lacecap ancestor. The inflorescence type is a monogenic, dominant-recessive trait (Uemachi and Okumura, 2012). Thus, we propose that all “Wädenswil” lacecap cultivars carry at least one lacecap allele from the wild type ancestor.

The “Wädenswil” pedigree includes diploid and triploid cultivars (Zonneveld, 2004; Jones et al., 2007). Diploid *H. macrophylla* varieties contain *2n* = *2x* = 36 chromosomes, while triploid varieties possess *2n* = *3x* = 54 chromosomes. Thereby, the 2C DNA content of *H. macrophylla* ranges from 3.85 to 4.97 pg for diploid varieties and from 6.48 to 7.27 pg for triploid varieties (Cerbah et al., 2001; Zonneveld, 2004; Jones et al., 2007; Gürtler et al., 2013; Alexander, 2017). Triploid hydrangeas develop larger organs and floral structures than diploids (Alexander, 2017). Furthermore, they are regarded to be more robust against biotic and abiotic stresses and are desired in breeding for cultivar selection. However, little is known about breeding triploid hydrangeas. As observed in other species, triploids can be generated through somatic fusion, sexual hybridization between reduced and unreduced gametes of diploid parents or sexual hybridization between a diploid and a tetraploid parent (Wang et al., 2016). To our knowledge, natural tetraploids of *H. macrophylla* have not been identified until now, although tetraploid *H. macrophylla* plants can be generated by artificially induced autoploidization as shown for the diploid cultivars ‘Adria’ and ‘Libelle’ (Gürtler et al., 2013). Recently, the production of triploid hydrangeas via unreduced male gametes was described (Alexander, 2017). Crosses between the diploid cultivars ‘Princess Juliana’ and ‘Trophee’ as well as ‘Zaunkönig’ and ‘Princess Juliana’ resulted in triploid and diploid F_1_ plants, while the reciprocal cross and a cross with ‘Zaunkönig’ as male parent resulted in only diploid offspring. Alexander (2017) hypothesized that the triploids resulted from sexual autopolyploidy, which was most likely caused by genotype-specific production of unreduced male gametes.

It is unknown whether interploid crosses between diploid and triploid hydrangeas can be used to develop triploid varieties. As observed in other species, triploids tend to be sterile and seedless due to meiotic errors (Sattler et al., 2016; Wang et al., 2016). Due to this, triploids are usually excluded from systematic breeding programs. However, DNA content data of “Wädenswil” cultivars (Zonneveld, 2004; Jones et al., 2007) revealed, that triploid hydrangeas have been developed in breeding to a considerable extent. Apparently interploid crosses between diploid and triploid genotypes have been successfully performed, although probably unwittingly. In order to prove this assumption, we collected 1–6 plants of 43 *H. macrophylla* cultivars from different sources. We determined their DNA content and inflorescence type to characterize this germplasm collection. We found several cultivars that included diploid and triploid plants or plants that showed either the lacecap or mophead inflorescence type. In order to clarify these mixes, we performed an SSR marker analysis. Within this study, we present (i) a systematic DNA content screening of 120 plants that represent in total 43 cultivars, including nearly all “Wädenswil” cultivars, (ii) a genotype identification based on SSR marker fingerprints, and (iii) a cultivar-specific genotype identification based on the molecular reconstruction of the “Wädenswil” pedigree.

## Materials and methods

### Plant material

We studied 1–6 plants of 43 cultivars, resulting in 120 plants (Table 1). This collection included 1–5 plants of 26 “Wädenswil” lacecap cultivars and 1–6 plants of ‘Tödi’, ‘Bodensee’, ‘Enziandom’, ‘Glärnisch’, and ‘Hörnli’, which were used as crossing partners to develop “Wädenswil” cultivars (Meier, 1990). No plants were found for the crossing partners ‘Speer’, Nr. 1703, Nr. 1192, and Nr. 254. In addition, we included 11 cultivars with 1 to 6 plants, which were already available in Europe before the “Wädenswil” breeding program started (Möhring et al., 1956). Finally, we included ‘Bela’, which was assumed to be a spontaneous, somatic mophead mutant of the “Wädenswil” cultivar ‘Blaumeise’ (Guérin, 2002). Forty-seven plants were provided by the Botanical Garden Pirna-Zuschendorf of the Dresden University of Technology, a member of the German Gene Bank for Ornamental Plants. Seventy-three plants were provided by Kötterheinrich-Hortensienkulturen.

**Table 1:** DNA content, inflorescence type and SSR marker fingerprint of 120 *H. macrophylla* plants.

**Cultivar**		**Plant**	**2C DNA content [pg]**	**Inflorescence type**	**Binary index matrix of SSR markers** **#1_#2 ___#3__#4__#5____ #6____#7___#8 _____#9_____#10 ___#11____#12**	**SSR fingerprint**	**Corrected name**
(01)	Bachstelze^a^	1	4.33	lacecap	01_10_0001_011_011_00100_00001_1001_00100_0001000_00100_100100	G01	Bachstelze
(02)	Bela^d^	1	6.54	mophead	11_11_0001_011_100_01101_00001_1001_00100_0001000_11001_000001	G02	Bela
		2	6.59	mophead	11_11_0001_011_100_01101_00001_1001_00100_0001000_11001_000001	G02	Bela
(03)	Benelux^c^	1	n.d.	mophead	11_11_0001_011_110_01000_00101_1001_10100_0101000_11000_010001	G03	Benelux
(04)	Bergfink^a^	1	6.70	lacecap	01_01_0001_001_100_01100_00001_1001_00101_0001010_01001_001000	G04	Bergfink
		2	6.66	lacecap	01_11_0001_001_110_01100_00101_1001_01101_0001010_11001_001000	G05	Buchfink
(05)	Blaukehlchen^a^	1	4.50	lacecap	01_11_0001_010_100_01000_00001_0001_00100_0001000_01001_001000	G06	Blaukehlchen
(06)	Bläuling^a^^,^^b^	1	4.40	n.d.	11_10_0001_001_110_01100_00001_0001_01100_0101000_11000_100001	G07	Bläuling
		2	4.45	lacecap	11_10_0001_001_110_01100_00001_0001_01100_0101000_11000_100001	G07	Bläuling
		3	4.47	n.d.	11_10_0001_001_110_01100_00001_0001_01100_0101000_11000_100001	G07	Bläuling
		4	4.48	lacecap	11_10_0001_001_110_01100_00001_0001_01100_0101000_11000_100001	G07	Bläuling
		5	6.50	lacecap	11_11_0001_011_100_01101_00001_1001_00100_0001000_11001_000001	G02	Blaumeise
(07)	Blaumeise^a^	1	6.49	lacecap	11_11_0001_011_100_01101_00001_1001_00100_0001000_11001_000001	G02	Blaumeise
		2	6.49	lacecap	11_11_0001_011_100_01101_00001_1001_00100_0001000_11001_000001	G02	Blaumeise
(08)	Bodensee^b^	1	4.33	n.d.	11_10_0001_001_101_01001_00100_1001_00100_0001000_10100_001001	G08	Bodensee I
		2	4.40	mophead	11_10_0001_011_100_01001_00001_1001_10100_1000001_10000_001001	G09	Bodensee II
		3	4.46	mophead	11_10_0001_011_100_01001_00001_1001_10100_1000001_10000_001001	G09	Bodensee II
		4	4.36	mophead	01_10_0001_001_101_01000_00101_0001_00100_0001001_10000_011000	G10	Unknown 1
		5	6.54	mophead	11_11_0001_011_100_01101_00001_1001_00100_0001000_11001_000001	G02	Bela
		6	6.55	mophead	11_11_0001_011_100_01101_00001_1001_00100_0001000_11001_000001	G02	Bela
(09)	Buchfink^a^	1	6.71	lacecap	01_11_0001_001_110_01100_00101_1001_01101_0001010_11001_001000	G05	Buchfink
		2	6.72	lacecap	01_11_0001_001_110_01100_00101_1001_01101_0001010_11001_001000	G05	Buchfink
		3	6.74	lacecap	01_11_0001_001_110_01100_00101_1001_01101_0001010_11001_001000	G05	Buchfink
(10)	Buntspecht^a^	1	4.35	lacecap	01_11_0001_001_110_00100_00001_1001_00100_0001010_10001_001000	G11	Buntspecht
		2	4.39	lacecap	01_11_0001_001_110_00100_00001_1001_00100_0001010_10001_001000	G11	Buntspecht
		3	6.60	n.d.	01_01_0001_001_100_01100_00001_1001_00101_0001010_01001_001000	G04	Bergfink
		4	6.68	lacecap	01_01_0001_001_100_01100_00001_1001_00101_0001010_01001_001000	G04	Bergfink
(11)	Eisvogel^a^	1	6.61	lacecap	11_11_0001_001_100_01001_00001_1011_10100_0001000_11001_000001	G12	Eisvogel
		2	6.57	lacecap	11_11_0001_011_100_01101_00001_1001_00100_0001000_11001_000001	G02	Blaumeise
(12)	Elster^a^	1	4.38	n.d.	01_10_0001_010_110_00100_00001_1100_01100_0001000_00100_101000	G13	Elster
		2	4.45	lacecap	01_10_0001_010_110_00100_00001_1100_01100_0001000_00100_101000	G13	Elster
(13)	Enziandom^b^	1	4.39	mophead	01_11_0001_001_110_01000_00101_1001_00100_0001000_10100_001001	G14	Unknown 2
		2	6.62	mophead	11_10_0001_001_101_01001_00001_1001_10100_0001100_01100_001001	G15	Unknown 3
		3	6.57	mophead	11_10_0001_001_101_01001_00001_1001_10100_0001000_01100_001001	G16	Unknown 4
		4	6.66	mophead	01_10_0001_001_110_01000_00001_0001_10100_0001100_10000_011001	G17	Unknown 5
(14)	Fasan^a^	1	6.59	lacecap	11_11_0001_011_101_01001_00001_0011_10001_0001011_10001_001001	G18	Unknown 6
		2	6.60	n.d.	11_11_0001_011_101_01001_00001_0011_10001_0001011_10001_001001	G18	Unknown 6
		3	4.34	lacecap	01_10_0001_011_100_01100_00001_1001_00100_0001000_10001_001000	G19	Fasan I
		4	4.41	lacecap	01_10_0001_001_100_01000_00001_1001_00100_0001000_10001_001100	G20	Fasan II
		5	4.43	mophead	11_11_0001_101_110_01001_10100_0001_10001_0001100_11000_000001	G21	Unknown 7
(15)	Flamingo^a^	1	4.51	lacecap	01_11_0001_001_101_01000_00001_0001_00100_0001000_10001_001000	G22	Flamingo I
		2	4.41	lacecap	01_11_0001_010_100_01000_00001_0001_00100_0001000_10001_001000	G23	Flamingo II
		3	4.46	mophead	01_10_0001_001_100_01000_00100_0001_00100_0001000_10000_000001	G24	Unknown 8
		4	4.52	mophead	01_10_0001_001_100_01000_00100_0001_00100_0001000_10000_000001	G24	Unknown 8
(16)	Geoffrey Chadbund^c^	1	4.41	lacecap	01_10_0001_001_110_00101_11001_0001_00001_0001000_01100_010100	G25	Geoffrey Chadbund
		2	4.41	lacecap	01_10_0001_001_110_00101_11001_0001_00001_0001000_01100_010100	G25	Geoffrey Chadbund
(17)	Gimpel^a^	1	4.42	lacecap	11_10_0001_011_100_01001_00101_0001_01100_0101000_10100_001001	G26	Gimpel
		2	4.43	lacecap	11_10_0001_011_100_01001_00101_0001_01100_0101000_10100_001001	G26	Gimpel
(18)	Glärnisch^b^	1	4.42	mophead	01_11_0001_011_110_00100_00101_1001_00100_0001000_10000_001001	G27	Glärnisch
(19)	Grasmücke^a^	1	4.37	lacecap	01_11_0001_011_uuu_01100_00001_0001_00101_0001010_10001_001000	G28	Grasmücke
		2	n.d.	n.d.	01_11_0001_011_101_01100_00001_0001_00101_0001010_10001_001000	G28	Grasmücke
		3	4.42	lacecap	01_10_0001_110_100_00110_00011_0101_10000_0000011_00100_000011	G29	Unknown 9
		4	4.46	lacecap	01_10_0001_110_100_00110_00011_0101_10000_0000011_00100_000011	G29	Unknown 9
(20)	Hörnli^b^	1	4.44	mophead	01_11_0001_001_100_01000_00101_0001_00100_0101000_11000_011000	G30	Hörnli
		2	4.46	mophead	01_11_0001_001_100_01000_00101_0001_00100_0101000_11000_011000	G30	Hörnli
		3	4.49	mophead	01_11_0001_001_100_01000_00101_0001_00100_0101000_11000_011000	G30	Hörnli
(21)	Kardinal^a^	1	6.55	lacecap	11_01_0001_011_100_01001_00001_0011_10001_0001010_10001_001001	G31	Unknown 10
		2	6.61	lacecap	01_11_0001_001_100_01100_00101_1001_01101_0001010_10001_001000	G32	Kardinal or Rotdrossel
		3	6.62	lacecap	01_11_0001_001_100_01100_00101_1001_01101_0001010_10001_001000	G32	Kardinal or Rotdrossel
		4	6.79	n.d.	01_11_0001_001_100_01100_00101_1001_01101_0001010_10001_001000	G32	Kardinal or Rotdrossel
		5	6.81	lacecap	01_11_0001_001_100_01100_00101_1001_01101_0001010_10001_001000	G32	Kardinal or Rotdrossel
(22)	Libelle^a^^,^^b^	1	4.38	n.d.	01_10_0001_011_110_00100_00001_0101_01100_0001000_01100_100100	G33	Libelle
		2	4.44	lacecap	01_10_0001_011_110_00100_00001_0101_01100_0001000_01100_100100	G33	Libelle
		3	4.61	n.d.	01_10_0001_011_110_00100_00001_0101_01100_0001000_01100_100100	G33	Libelle
		4	4.40	lacecap	01_10_0001_011_011_00100_00001_1001_00100_0001000_00100_100100	G01	Bachstelze
(23)	Mariesii^c^	1	6.70	lacecap	01_10_0001_011_110_01100_10100_1001_10101_0001001_11100_011000	G34	Mariesii
		2	4.53	n.d.	01_10_0001_011_100_01000_00100_0011_10100_0001001_10100_011000	G35	Mariesii Perfecta
		3	4.55	n.d.	01_10_0001_011_100_01000_00100_0011_10100_0001001_10100_011000	G35	Mariesii Perfecta
(24)	Mariesii Grandiflora^c^	1	4.32	lacecap	01_11_1001_011_100_10100_01000_0101_10100_1001000_10010_101000	G36	Mariesii Grandiflora
(25)	Mariesii Lilacina^c^	1	4.16	lacecap	01_11_0101_001_101_01100_00001_0010_00100_0100001_00110_001010	G37	Mariesii Lilacina
		2	4.18	lacecap	01_11_0101_001_101_01100_00001_0010_00100_0100001_00110_001010	G37	Mariesii Lilacina
		3	4.28	lacecap	01_11_0101_001_101_01100_00001_0010_00100_0100001_00110_001010	G37	Mariesii Lilacina
		4	4.31	n.d.	01_11_0101_001_101_01100_00001_0010_00100_0100001_00110_001010	G37	Mariesii Lilacina
		5	4.33	n.d.	01_11_0101_001_101_01100_00001_0010_00100_0100001_00110_001010	G37	Mariesii Lilacina
		6	4.39	n.d.	01_11_0101_001_101_01100_00001_0010_00100_0100001_00110_001010	G37	Mariesii Lilacina
(26)	Mariesii Perfecta^c^	1	4.53	lacecap	01_10_0001_011_100_01000_00100_0011_10100_0001001_10100_011000	G35	Mariesii Perfecta
		2	4.54	n.d.	01_10_0001_011_100_01000_00100_0011_10100_0001001_uuuuu_011000	G35	Mariesii Perfecta
(27)	Mathilde Gütges^c^	1	4.44	mophead	11_10_0001_001_010_01001_00101_1001_00100_0001000_10100_001001	G38	Mathilde Gütges I
		2	n.d.	n.d.	11_10_0001_001_101_01000_00100_1001_00100_0001000_10100_001001	G39	Mathilde Gütges II
(28)	Madame Emile Mouillère^c^	1	4.50	mophead	01_11_0001_010_101_01000_00001_0011_10100_0001000_10100_101000	G40	Madame Emile Mouillère
(29)	Möwe^a^	1	6.48	lacecap	11_10_1001_011_110_01100_00001_0001_00101_0001010_11001_001011	G41	Möwe
		2	6.61	lacecap	11_10_1001_011_110_01100_00001_0001_00101_0001010_11001_001011	G41	Möwe
		3	4.41	n.d.	01_10_0001_011_110_00101_00001_0001_01100_0001010_01001_101000	G42	Unknown 11
(30)	Mücke^a^	1	4.45	lacecap	01_10_0001_011_100_01100_00100_0011_10100_0001001_10100_011000	G43	Mücke I
		2	6.69	mophead	11_11_1001_001_110_01101_00001_0001_10100_0001000_11100_001001	G44	Unknown 12
		3	4.45	lacecap	01_10_0001_011_111_00101_00001_0001_01100_0001010_01001_101000	G45	Mücke II
(31)	Nachtigall^a^	1	6.51	lacecap	11_11_0001_011_100_01001_00001_1011_10101_0001010_11001_000001	G46	Nachtigall
		2	6.52	n.d.	11_11_0001_011_100_01001_00001_1011_10101_0001010_11001_000001	G46	Nachtigall
		3	4.53	lacecap	01_10_0001_011_100_01100_00001_0001_00101_0001010_10001_001001	G47	Rotkehlchen
(32)	Nikko Blue^c^	1	4.47	mophead	11_11_0001_101_110_01000_01001_0001_10001_0001100_11000_001001	G48	Nikko Blue I
		2	3.95	lacecap	01_11_0011_011_101_00100_11000_0110_00010_1010000_00010_001100	G49	Nikko Blue II
(33)	Papagei^a^	1	4.46	lacecap	01_10_0001_011_110_00100_00001_0101_01100_0001000_01100_100100	G33	Libelle
		2	4.36	n.d.	01_11_0001_001_110_01100_00101_1000_00101_0001000_10001_001001	G50	Unknown 13
(34)	Pfau^a^	1	4.44	lacecap	01_10_0001_011_001_01100_00001_0001_00100_0101000_10001_101000	G51	Pfau I
		2	4.52	lacecap	01_10_0001_011_001_01100_00001_0001_00100_0101000_10001_101000	G51	Pfau I
		3	6.65	lacecap	11_11_0001_001_100_01001_00001_1011_10100_0001000_11001_000001	G12	Eisvogel
		4	4.40	mophead	11_10_1001_001_100_01001_00001_0001_00100_0001000_11000_001000	G52	Pfau II
(35)	Renate Steiniger^c^	1	4.41	mophead	11_10_0001_011_100_01001_00001_1000_00100_0001001_10000_001100	G53	Renate Steiniger I
		2	4.51	mophead	11_10_0001_011_100_01001_00001_1000_00100_0001001_10000_001100	G53	Renate Steiniger I
		3	4.53	mophead	11_10_0001_011_100_01001_00001_1000_00100_0001001_10000_001100	G53	Renate Steiniger I
		4	4.47	n.d.	11_10_0001_011_100_01001_00001_1000_00100_0001001_10000_001000	G54	Renate Steiniger II
(36)	Rotdrossel^a^	1	6.66	lacecap	01_11_0001_001_100_01100_00101_1001_01101_0001010_10001_001000	G32	Rotdrossel or Kardinal
		2	6.73	lacecap	01_11_0001_001_100_01100_00101_1001_01101_0001010_10001_001000	G32	Rotdrossel or Kardinal
(37)	Rotkehlchen^a^^,^^b^	1	4.45	lacecap	01_10_0001_011_100_01100_00001_0001_00101_0001010_10001_001001	G47	Rotkehlchen
		2	6.47	lacecap	11_11_0001_011_100_01101_00001_1001_00100_0001000_11001_000001	G02	Blaumeise
		3	6.53	lacecap	11_11_0001_011_100_01101_00001_1001_00100_0001000_11001_000001	G02	Blaumeise
(38)	Rotschwanz^a^	1	4.46	lacecap	01_10_0001_011_100_00101_00101_1001_00101_0001000_00101_001000	G55	Rotschwanz I
		2	4.41	lacecap	01_11_0001_011_100_00101_00101_0001_00101_0001000_00101_001000	G56	Rotschwanz II
(39)	Taube^a^^,^^b^	1	6.57	lacecap	11_11_0001_011_100_01101_00001_1001_00100_0001000_11001_000001	G02	Blaumeise
(40)	Tödi^b^	1	4.54	mophead	11_10_1001_001_101_01100_00001_1001_10100_0001000_10100_001100	G57	Tödi
		2	6.49	mophead	01_11_0001_001_100_01001_00001_0001_00101_0001000_10100_001101	G58	Unknown 14
(41)	Veitchii^c^	1	4.21	lacecap	01_11_1001_011_110_10100_11000_0101_10100_1001000_10010_101000	G59	Veitchii
		2	4.22	lacecap	01_11_1001_011_110_10100_11000_0101_10100_1001000_10010_101000	G59	Veitchii
		3	4.23	lacecap	01_11_1001_011_110_10100_11000_0101_10100_1001000_10010_101000	G59	Veitchii
		4	4.45	n.d.	01_10_0001_011_110_00100_00001_0101_01100_0001000_01100_100100	G33	Libelle
(42)	Zaunkönig^a^	1	4.41	lacecap	01_11_0001_011_101_01100_00001_0001_01100_0101000_01001_101000	G60	Zaunkönig
		2	4.44	lacecap	01_11_0001_011_101_01100_00001_0001_01100_0101000_01001_101000	G60	Zaunkönig
(43)	Zeisig^a^^,^^b^	1	4.44	lacecap	01_11_0001_011_101_01000_00001_0001_00101_0001010_10001_001000	G61	Zeisig
		2	4.54	lacecap	01_11_0001_011_101_01000_00001_0001_00101_0001010_10001_001000	G61	Zeisig
		3	4.46	lacecap	01_11_0001_001_100_01000_00001_1000_00100_0001010_10001_000001	G62	Unknown 15

a “Wädenswil” cultivar;

b crossing partner in the “Wädenswil” breeding program;

c Cultivar that existed already before the “Wädenswil” breeding program started and which might be related or identical to unknown crossing partners;

d *mophead mutant of ‘Blaumeise’; n.d., not determined; SSR marker fingerprints are presented as binary code with 0 (absent) and 1 (present) alleles based on the order of SSR markers 1–12 and the corresponding fragment lengths listed in Table 2*.

Plants were cultivated in 17 cm pots filled with Einheitserde® CL Hortensien blau on tables in a frost-free greenhouse of the Leibniz Institute of Vegetable and Ornamental Crops in Erfurt, Germany, without additional light supply. Plants were fertilized with Universol® blue 0.1% (Everris International BV) and irrigated as necessary. At the beginning of July all plants were pruned.

### Phenotyping

The inflorescence type was recorded in 2016 and 2017, when the plants were in full bloom. *Hydrangea* plants show a distinct inflorescence type: If decorative flowers are present only at the periphery of the inflorescences, then the plant is recorded as lacecap. If all non-decorative flowers are covered by decorative flowers, then the plant is recorded as mophead.

### Flow cytometry

2C DNA content was determined by flow cytometry according to Dolezel et al. (2007) with slight modifications. We used *Pisum sativum* L. ‘Ctirad’ with a 2C DNA content of 9.09 pg as internal standard. About 0.3 and 0.9 cm^2^ of young leaves of sample and standard were chopped with a razor blade for 30–60 s in a plastic petri dish containing 1 ml Galbraith's buffer (45 mM MgCl_2_, 20 mM MOPS, 30 mM sodium citrate, 0.1% (v/v) Triton X-100, pH 7) freshly supplemented with 50 μg/ml propidium iodide, 50 μg/ml RNAse A and 1% (w/v) PVP 25. The homogenate was mixed by pipetting, passed through a 30 μm CellTrics filter (Partec) and analyzed using a Partec CyFlow Space analyzer with a 488 nm blue solid state laser at a flow rate of 0.1 μl/s. Data analysis was performed using the software FloMax version 2.70 (Quantum Analysis GmbH). For each sample-standard-mixture, about 10,000 nuclei were analyzed, which yielded about 5,000 nuclei per sample. High quality peaks were determined at CV < 4%. The 2C DNA content of each sample was calculated in relation to the 2C DNA content of the standard as follows: 2C DNA content sample = mean fluorescence value of sample ^*^ 9.09 pg / mean fluorescence value of *P. sativum*.

### Chromosome counting

Based on flow cytometric data, 3 diploid and 2 triploid plants were selected for chromosome counts. Root tips were incubated in 2 mM 8-hydroxyquinoline for 3 h at room temperature and fixed in 3:1 ethanol:acetic acid for at least 24 h. Subsequently, the root tips were washed 10 min in aqua dest. and macerated for 40 min at 37°C in an enzymatic solution containing 4% cellulose R10 (Duchefa), 1% pectolyase Y-23 (Duchefa), 75 mM KCl and 7.5 mM Na_2_EDTA at pH 4. Afterwards, the root tips were washed in aqua dest., transferred onto a slide, covered with 6 μl 45% acetic acid and carefully squeezed under a cover slip. Then, the cover slip was removed by freezing the slide in liquid nitrogen. After drying the slides, chromosomes were stained with DAPI using VECTASHIELD® HardSet™ Antifade Mounting Medium with DAPI (VECTOR Laboratories, Inc.) according to the manufacturer's protocol. Chromosomes were visualized using a fluorescence microscope with 360 nm excitation and 460 nm emission filter. Chromosome counts were made from 5 metaphase cells per examined cultivar.

### DNA extraction and marker analysis

Genomic DNA was extracted from young leaves. About 100 mg shock-frozen leaf samples were homogenized using Precellys®24 with the Cryolys® cooling module (Bertin Technologies S.A.S.). DNA was isolated using the DNeasy® Plant Mini Kit (QIAGEN) according to the manufacturer's protocol. DNA was eluted with aqua dest. DNA concentration was measured using the NanoDrop 2000c (Thermo Scientific).

We used 11 Simple Sequence Repeat (SSR) markers that were already used to estimate the genetic diversity for the genus *Hydrangea* (Rinehart et al., 2006; Reed and Rinehart, 2007). The characterization of these SSR loci based on 114 *H. macrophylla* taxa was published by Reed and Rinehart (2007). In addition, we developed one SSR marker based on an RNAseq contig sequence published by Chen et al. (2015). These SSR markers detected between 2 and 7 alleles per locus in our germplasm collection, on average 4.25 alleles per locus. The marker information and corresponding primer sequences are given in Table 2. All primers were obtained from Metabion International AG. Polymerase chain reaction (PCR) assays were done in a total volume of 12.5 μl containing 5 ng DNA, 1x PCR buffer including MgCl_2_ (Metabion International AG), 0.2 mM dNTPs (Metabion International AG), 0.2 μM unlabeled forward and reverse primers, additionally 0.004 μM primer labeled with IRD700 or 0.006 μM primer labeled with IRD800 (Metabion International AG), and 0.02 U mi-Taq DNA polymerase (Metabion International AG). The PCR conditions were 3 min at 94 °C, 35 cycles of 30 s at 94 °C, 30 s at 60 °C and 30 s at 72 °C, and finally 5 min at 72 °C. PCR fragment lengths were determined by polyacrylamide gel electrophoresis according to Borchert and Gawenda (2010). For this, we mixed 5 μl IRD 700 PCR product, 5 μl IRD 800 PCR product and 90 μl pararosaniline loading dye and separated PCR fragments together with a 50–700 bp sizing standard (LI-COR Biosciences) on a 6.5% KB^Plus^ gel matrix (LI-COR Biosciences) using a LI-COR 4300 DNA analyzer (LI-COR Biosciences). Data analysis was performed with the software program SAGA 3.3 (LI-COR Biosciences). PCR fragment lengths were determined in relation to the 50–700 bp sizing standard (LI-COR Biosciences). For each genotype, SSR marker alleles were recorded in a binary code as 1 (present) and 0 (absent).

**Table 2:** SSR markers used in this study.

**Marker number**	**Marker name**	**SSR motif**	**Forward primer**	**5′ to 3′ sequence**	**Tm [°C]**	**Reverse primer**	**5′ to 3′ sequence**	**Tm [°C]**	**Number of detected alleles**	**Allele-specific PCR fragment lengths [bp]**
#01	STAB071_072^a^	(TGA)_8_	A033	CTTGTCACAAACCGTCTTTCC	60	A034	GGAAATGGGGGATTTTGATTTCG	61	2	146/140
#02	STAB379_380^a^	(ATC)_6_	A077	CATCTCAATGCCAAACCCTAAAC	61	A078	GGTTTTGTGATCTTCAGCTCTC	60	2	167/161
#03	STAB125_126^a^	(CTT)_4_	A007	CAGTATCTCTGCCCAATCGAG	61	A008	GATGACCAGAACGATGAGAATG	60	4	162/159/153/147
#04	STAB173_174^a^	(TCAGTT)_4_	A047	GTTGTGGTGTTGAAGATCTTCTG	61	A048	CGGTTCTTGATCTTCTTCGTG	60	3	180/174/168
#05	STAB317_318^a^	(AAG)_8_	A069	GTCAAGTAATCATGGGGTCAC	60	A070	GACAGCAACTCTTCATCAGTG	60	3	155/149/146
#06	STAB321_322^a^	(TCT)_7_	A015	CAATTTCACCCATTTGAGGCC	60	A016	GGACTTACAGTCGCCGAGC	60	5	156/153/150/147/138
#07	A029_A030^b^	(AG)_10_	A029	CCTAATCTCCTCACTCTCTTTC	60	A030	GCATTGGACGGACTCAATTGG	61	5	153/150/147/143/137
#08	STAB161_162^a^	(CAG)_7_	A043	GCAGAAGCGCGATGTCAATC	61	A044	CAGGATATTAGGAGATGGACTC	60	4	158/147/141/138
#09	STAB137_138^a^	(ATC)_10_	A041	GGTCTTCGGAAAACTCACATTC	60	A042	CGTTGAATTCTTGGTTACAGGC	60	5	150/147/143/138/135
#10	STAB227_228^a^	(TTC)_12_	A053	CTCCAGTTCCTTGATAGCATG	60	A054	CCTGAGAGTACGTACAGCAG	61	7	180/177/172/152/143/134/128
#11	STAB305_306^a^	(CAG)_8_	A065	CTAACTAGATCCAGACCAACAAC	61	A066	CACGATGGACCCATAAAAGGC	61	5	132/129/126/120/118
#12	STAB241_242^a^	(TTC)_10_	A057	GGAGACAATATTTCGTTCCAGTG	61	A058	GCTTCTAGTTTTGTCAACAGCAG	61	6	125/119/116/113/107/101

a *Marker developed by Reed and Rinehart (2007), modified in this study by choosing other primer binding sites*.

b *Marker developed in this study based on a RNAseq contig sequence published by Chen et al. (2015)*.

## Results

### Variability of phenotype and ploidy level

We studied 120 plants that belonged to 43 cultivars according to their labels (Table 1). These cultivars included all 26 “Wädenswil” lacecap cultivars as well as ‘Tödi’, ‘Bodensee’, ‘Enziandom’, ‘Glärnisch’, and ‘Hörnli’, which were used as crossing partners to develop “Wädenswil” lacecap cultivars (Meier, 1990). Furthermore, this collection included 11 cultivars available prior to the “Wädenswil” breeding program and also ‘Bela’, an assumed spontaneous mophead mutant of ‘Blaumeise’ (Guérin, 2002). All cultivars were represented by 1–6 plants from different sources.

**Figure 1:**
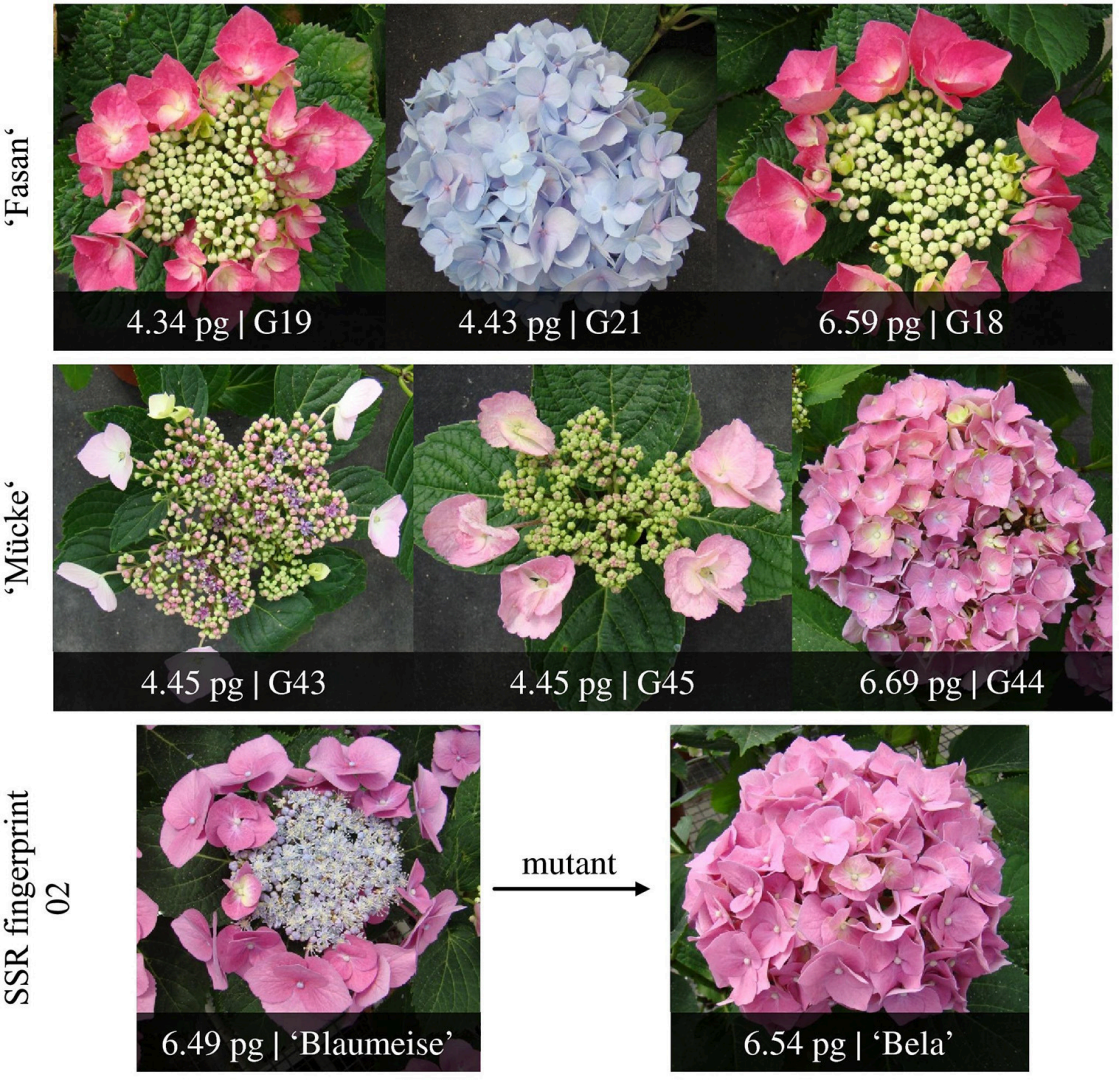
Examples of plants that varied in inflorescence type, 2C DNA content or SSR marker fingerprint.

Firstly, we recorded the inflorescence type. In total, 69 plants developed lacecap inflorescences, while 29 plants produced mophead inflorescences (Table 1). The inflorescence type of the remaining 22 plants was not determined, because these plants did not flower. Interestingly, 5 cultivars (namely ‘Fasan’, ‘Mücke’, ‘Pfau’, ‘Flamingo’, ‘Nikko Blue’) contained plants that produced either lacecap or mophead inflorescences (Figure 1). These varying inflorescence types may be the result of mixed plants or spontaneous mutations at the inflorescence locus.

Secondly, we determined the 2C DNA content of all plants by flow cytometry, in order to identify diploid and triploid plants. In total, we detected 78 plants, whose 2C DNA content ranged from 3.95 to 4.61 pg with an average of 4.42 ± 0.10 pg. For 39 plants, we measured 2C DNA contents from 6.47 to 6.81 pg, on average 6.61 ± 0.09 pg (Table 1). We counted 36 chromosomes for ‘Bläuling’, ‘Bodensee’, and ‘Libelle’, whose 2C DNA content ranged from 4.40 to 4.45 pg. In contrast, 54 chromosomes were counted for ‘Blaumeise’ and ‘Bela’ with 2C DNA contents of 6.50 and 6.59 pg, respectively. In addition, Jones et al. (2007) reported 36 chromosomes for ‘Veitchii’ and 54 chromosomes for ‘Nachtigall’ and ‘Taube’, whose 2C DNA contents was determined with 4.22, 6.51, and 6.57 pg, respectively. Thus, our *Hydrangea* collection included 78 putatively diploid and 39 putatively triploid plants. However, aneuploids cannot be excluded, because flow cytometry allows only an approximation of chromosome numbers. Surprisingly, 12 out of 43 cultivars included diploid as well as triploid plants.

### SSR fingerprinting identified up to four different genotypes within one cultivar

Cultivars of *H. macrophylla* are vegetatively propagated. Therefore, all clones of a cultivar have the same genotype and should display the same chromosome number and inflorescence type. In order to test whether these varying plants have identical genotypes, we performed an SSR marker analysis using 12 polymorphic SSR markers. These markers detected 2–7 different alleles per marker and gave in total 51 different alleles within the 120 plants (Table 1). Using these 12 SSR markers, we identified 18 cultivars whose plants showed a unique, cultivar-specific SSR marker fingerprint. However, 25 out of 43 cultivars comprised 2–4 different genotypes based on the SSR marker fingerprints. In total, we detected 62 distinct genotypes (Table 1), although only 43 genotypes (one genotype per cultivar) were expected.

Our 12 SSR markers were unable to differentiate between ‘Blaumeise’ and ‘Bela’ nor ‘Kardinal’ and ‘Rotdrossel’. This result was expected for ‘Blaumeise’ and ‘Bela’, because ‘Bela’ is a mophead mutant of ‘Blaumeise’, which we confirmed now at molecular level. It is unlikely that any of our used SSR markers is able to detect this specific mutation. Thus, ‘Blaumeise’ and ‘Bela’ have an identical SSR marker fingerprint, but ‘Blaumeise’ develops lacecap inflorescences whereas ‘Bela’ produces mophead inflorescences. In contrast, ‘Kardinal’ and ‘Rotdrossel’ should display different genotypes as they originated from different crosses. The screened plants of ‘Kardinal’ and ‘Rotdrossel’ may belong to the same genotype and the other cultivar is missing in our collection. Alternatively, the marker number could be too low to detect genotype-specific polymorphisms. The probability that ‘Kardinal’ and ‘Rotdrossel’ show the identical fingerprint is *P* = 2.291E-06 based on the frequency of the 12 different SSR marker fingerprints within the triploid genotype pool analyzed in this study. Furthermore, all plants of ‘Kardinal’ and ‘Rotdrossel’ have a very similar plant phenotype. To confirm the hypothesis that all plants of ‘Kardinal’ and ‘Rotdrossel’ belong to the same genotype, more plants and markers should be analyzed.

For 13 genotypes, we detected up to 3 alleles at various marker loci. Three alleles at one locus indicate triploidy, which we confirmed for 11 out of 13 genotypes by flow cytometry. However, the genotypes G25 and G45 showed 3 alleles at marker loci 7 and 5, respectively, but 2C DNA contents of 4.41 and 4.45 pg, which suggests diploidy. Since we also detected these alleles independently in other plants, they were not rejected as unspecific PCR fragments, but might indicate duplicated chromosomal regions.

### Reconstruction of the “Wädenswil” pedigree on molecular level

**Figure 2:**
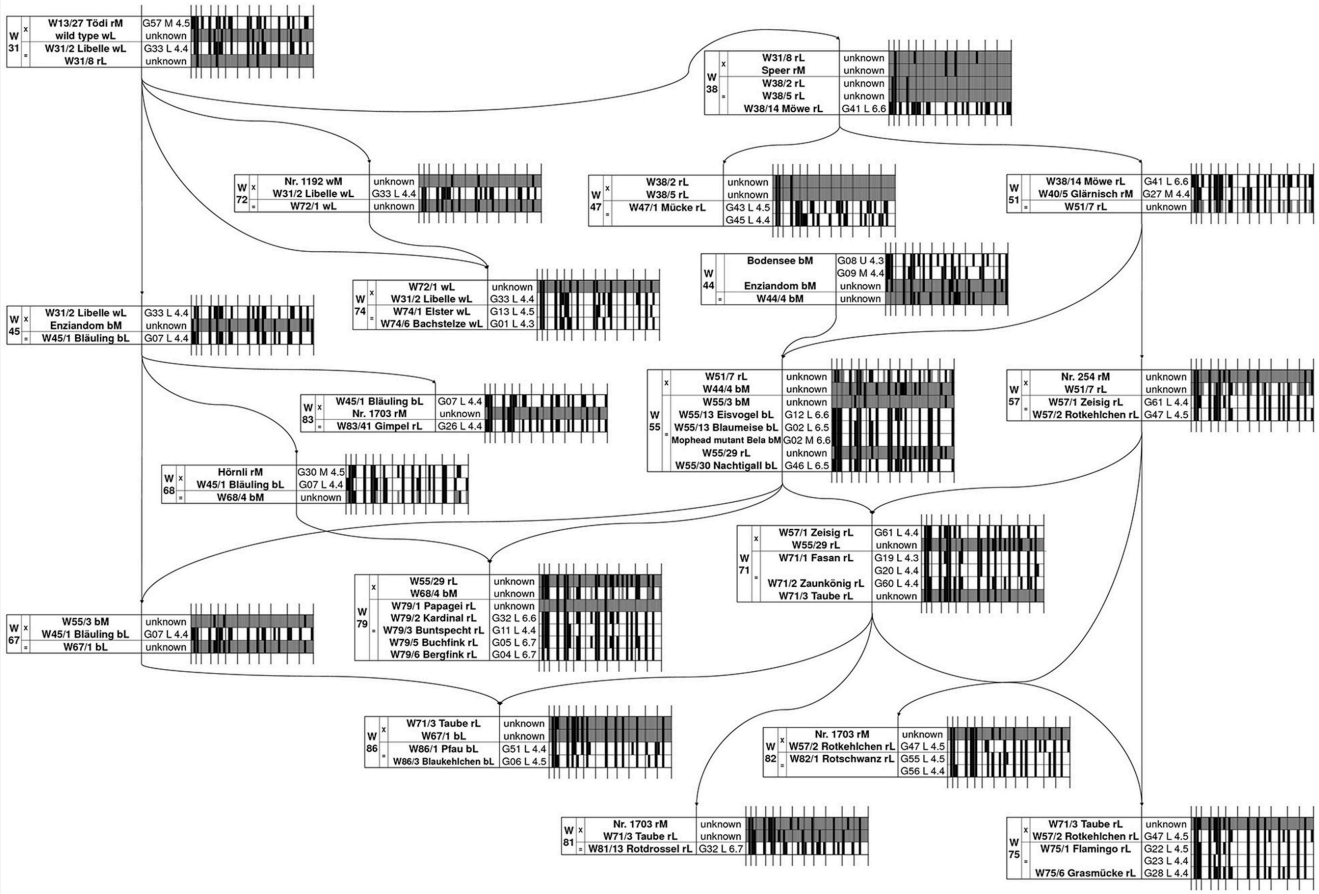
Reconstruction of the “Wädenswil” pedigree of lacecap cultivars of *H. macrophylla* according to Meier (1990) based on 12 SSR markers. The barcode tables show schematically the fingerprints of the SSR markers 1–12 as given in the binary index matrix in Table 1. The detected 51 SSR marker alleles are given as small bars as alleles • present, ◦ absent, or • allele configuration unknown at this position. Wxx/x, Wädenswil cross number/plant number; Gxx_M/L/U_x.x, SSR fingerprint _ inflorescence type _ 2C DNA content in pg; M, mophead; L, lacecap; U, unknown inflorescence type; r, red/pink; b, blue; w, white flower color.

All plants of a cultivar that varied in their inflorescence type or DNA content displayed different genotypes. Moreover, different genotypes were also detected between plants showing the same inflorescence type and ploidy level, e.g., ‘Bergfink’ or ‘Eisvogel’ (Table 1), therefore several plants must be labeled incorrectly. In order to identify the correct plant of a cultivar, we reconstructed the “Wädenswil” pedigree at molecular level. Based on the published “Wädenswil” pedigree (Meier, 1990), which is shown with slight modifications in Figure 2, we started the molecular reconstruction from the initial cross W31. We followed the inheritance of marker alleles from known parental genotypes to descendants or by predicting putative parental genotypes from known descendant genotypes based on two criteria: If a marker locus of a descendent is heterozygous, both parents (diploid or triploid) must contain at least one of these alleles. If a marker locus of a descendent is homozygous, both parents must contain at least this certain allele. For example, ‘Libelle’ was derived from cross W31 and is parent in cross W45 and W74. We detected two genotypes for ‘Libelle’, showing the SSR marker fingerprints G01 and G33, respectively. Only the allele configuration of G33 fitted into cross W31 with alleles derived from ‘Tödi’ (fingerprint G57), into cross W45 as parent of ‘Bläuling’ (fingerprint G07) and into cross W74 as back-cross parent of ‘Elster’ and ‘Bachstelze’, while several marker alleles of G01 failed. G01 was also detected as SSR marker fingerprint of ‘Bachstelze’. Thus, we assume that ‘Libelle’ with fingerprint G01 was misnamed and is actually ‘Bachstelze’. In cross W45, none of the four different ‘Enziandom’ genotypes contributed to alleles of ‘Bläuling’. We assume that either the correct ‘Enziandom’ is missing in our collection or that ‘Enziandom’ was not the crossing parent in W45. Furthermore, we were able to predict some allele configurations of the parental genotype based on our criteria. This predicted genotype can be used to identify the correct crossing partner in W45. Using this method, all cross combinations were analyzed. The complete molecular reconstruction of the “Wädenswil” pedigree is shown in Figure 2. We were able to assign the genotypes of 18 out of 26 “Wädenswil” cultivars. For ‘Fasan’, ‘Rotschwanz’, ‘Flamingo’, and ‘Mücke’ we could reduce the number of genotypes to two possible candidates, which both fitted into the pedigree. The SSR marker fingerprints of ‘Kardinal’ and ‘Rotdrossel’ fitted to both pedigree positions. Here, more plants and markers must be analyzed to identify the cultivar-specific genotype and fingerprint. In contrast, none of the SSR marker fingerprints, which were detected for different plants of ‘Enziandom’, ‘Papagei’, and ‘Taube’, fitted to the corresponding predicted allele configuration in the “Wädenswil” pedigree. Thus, the right genotypes of these three cultivars are missing in our collection.

### Differing ploidy levels within a cultivar are due to mix-up

We detected 12 out of 43 cultivars that included diploid and triploid plants. To prove, whether these diploids were derived from triploid precursors, we compared the allele configurations of triploid and diploid plants with the same cultivar name. If a chromosomal re-organization took place, all alleles of diploid plants would be present in the corresponding triploid precursor. However, none of the 12 cultivars that included diploid and triploid plants fulfilled this condition. Instead, we were able to assign several of these plants to other cultivars based on their SSR marker fingerprint, which then matched perfectly with the corresponding ploidy level (Table 1). Thus, all differing plants were mislabeled.

### Interploid crosses within the “Wädenswil” pedigree

In total, we detected at least 45 diploid and 15 triploid genotypes based on our marker analysis. 25 diploid and 7 triploid genotypes were attached to the “Wädenswil” pedigree (Figure 2). Our analysis identified cross W51 as an interploid cross between triploid ‘Möwe’ and diploid ‘Glärnisch’. Furthermore, the predicted genotypes of W44/4 and W55/29 contained up to 3 alleles at various marker loci, which suggests polyploidy. W44/4 and W55/29 were used as crossing partners in crosses W55, W71, and W79, which reveals 3 further interploid crosses in the “Wädenswil” pedigree. These crosses resulted in diploid or/and triploid offspring. Based on these findings, we assume that interploid crosses with triploids were successfully applied in *Hydrangea* breeding, resulting in diploid and triploid offspring.

In addition, crosses W38, W44, and W81 also gave triploid offspring. The mechanism that caused triploidy remains unclear, because most of the crossing partners and their DNA content are unknown.

## Discussion

By genotyping a set of 120 *H. macrophylla* plants with 12 SSR markers, we identified 25 out of 43 cultivars that included 2–4 plants with different SSR marker fingerprints that varied also with regard to DNA content and inflorescence type. In total, we detected at least 62 distinct genotypes instead of 43 expected ones. Approximately 36% of these plants carried a wrong cultivar name. Based on the published “Wädenswil” pedigree of lacecap hydrangeas, we were able to predict cultivar-specific SSR marker fingerprints and retrieved 18 out of 26 “Wädenswil” cultivars. Thus, genotyping allowed reducing the total number of plants to the extent of almost 52%, while 19 extra genotypes were identified in our germplasm collection.

Our study confirmed that genotyping with SSR markers is a powerful tool to identify and differentiate between cultivar-specific genotypes in clonally propagated plant species. Plants with the same genotype can be reduced to decrease the number of plants in germplasm collections. In contrast, plants with differing genotypes can be separated in the case of mix-ups, and can be assigned to the correct cultivar. This would be beneficial especially for gene banks. Gene banks conserve and document genetic resources and provide material and data to maintain genetic- and biodiversity (www.croptrust.org). The ability to sort and correct stock using SRR markers would be incredibly beneficial for gene banks. Around the world, about 1,750 gene banks hold more than 7.4 million seeds or plant tissues from thousands of crop species (Gruber, 2017). In October 2016, *Hydrangea* collections became part of the national Gene Bank for Ornamental Plants in Germany (Spellerberg, 2017). However, plant conservation is expensive and storage capacities are limited. To organize gene banks efficiently, redundancies in plant collections and misnamed plants must be avoided.

*Hydrangea* is a vegetatively propagated crop and each cultivar possesses a specific genotype that can be represented by only one plant. However, a strong mix-up of plants was detected in our plant collection, which partially represents two independent *Hydrangea* collections. Several mix-ups were clearly detectable by phenotype, for instance differing inflorescence types, but most were not obvious due to phenotypic similarity resulting from the close relationship of some *Hydrangea* cultivars. For example, the siblings ‘Blaumeise’, ‘Eisvogel’, and ‘Nachtigall’ are half-siblings of ‘Rotkehlchen’, while ‘Elster’ and ‘Bachstelze’ are backcross progenies of ‘Libelle’ (Meier, 1990). If the cultivar-specific differences are too small and outstanding characteristics are missing, cultivar identification by phenotype alone is critical and mix-ups can occur especially for old cultivars out of commercial production. Redundancies and mix-ups were also observed in germplasm collections of other species (Hokanson et al., 1998; Lund et al., 2003). Thus, we strongly recommend to document plants not only by phenotype but also by genotype, in order to identify mixed, to eliminate wrong and to reduce redundant plants. Furthermore, we propose to do plant descriptions only after genotype identification and to add these phenotypic and genotypic data to a public accessible data base.

For genotyping, the marker system and number of markers have to be individually chosen for each species according to the genetic diversity within the germplasm collection. In our plant collection, 12 SSR markers, which detected in total 51 different alleles, distinguished 62 out of putative 64 genotypes. SSR markers are very suitable to detect duplicates or misnamed plants (Hokanson et al., 1998; Lund et al., 2003). They are convenient for the genotyping of germplasm collections due to their locus-specific, co-dominant inheritance, high reproducibility and utilization in high throughput. Moreover, they are relatively cheap and data handling is easy. However, only a low number of loci are considered and the information about genetic diversity remains low. In times of *next generation sequencing*, resequencing of whole genomes and genotyping-by-sequencing are increasingly widespread and affordable (Lin et al., 2014; Cheng et al., 2016; Varshney et al., 2017). Although highly interesting with regard to genome-wide association analysis and pre-breeding research, genotyping-by-sequencing might be over-dimensioned for simple genotype identification. The number of markers used in this study is relatively low. However, genotype differentiation depends not only on the number of markers but also on the number of detected alleles and the allele frequencies within the germplasm collection. For systematic genotyping of *Hydrangea* gene bank collections, the marker number has to be established and this depends on the genetic diversity of the plant collection. This study provides a foundation for future *Hydrangea* genotype analyses using SSR markers. Although SSR markers seem to be old-fashioned in times of *next generation sequencing*, they fulfill the requirements of easy and reliable genotype identification nicely.

Based on the reconstruction of the “Wädenswil” pedigree, we identified four putative interploid crosses, which produced diploid or/and triploid progenies even in top-select quality. Thereby, triploids were used as seed and pollen parents. Crosses with triploids are considered to be difficult due to meiotic errors. Triploids are often sterile as observed for citrus, banana and watermelon (Wang et al., 2016), or they produce aneuploid gametes as observed in maize. Crosses between triploid and diploid maize plants resulted in aneuploid offspring with differing chromosome numbers and insufficient phenotype compared to the diploid and triploid parental plants (McClintock, 1929). In contrast to maize, cross experiments of triploid and tetraploid rose plants indicated that triploid roses were able to produce haploid and diploid male and female gametes (Van Huylenbroeck et al., 2005). The 2C DNA contents of verified “Wädenswil” cultivars gave no significant indication for aneuploidy, although we have DNA content variations in flow cytometric measurements in the range of ± 1 chromosome. However, this variation might be caused by natural variations in DNA content (Cerbah et al., 2001) or the technical error of flow cytometric measurements. In addition, all studied hydrangeas showed a normal, very attractive phenotype, which might be unlikely for aneuploid plants. The plants with SSR marker fingerprint G25 and G45 showed 3 alleles using the markers 7 and 5, respectively, although the corresponding plants were assumed to be diploid based on the corresponding 2C DNA content. All alleles were independently detected also in other plants and therefore cannot be excluded as unspecific PCR fragments. Three alleles in a nearly diploid background might indicate aneuploidy concerning single chromosomes. But before speculating about the generation of aneuploid hydrangeas, the corresponding chromosome numbers of these plants have to be determined and the identity of the 3 putative alleles must be verified in subsequent studies.

The “Wädenswil” pedigree gives clear indications, that triploid hydrangeas can be used for systematic breeding. Nevertheless, the generation of triploid, aneuploid and maybe tetraploid hydrangeas and their suitability in breeding programs requires further analyses including systematic cross experiments with various diploid and triploid varieties as well as cytogenetic analyses of meiosis.

## Author contributions

PH designed and performed experiments and analyzed the data, AH conceived the study and rose funding, CT supervised the study and wrote the manuscript. All authors revised the data, read and approved the final manuscript.

### Conflict of interest statement

The authors declare that the research was conducted in the absence of any commercial or financial relationships that could be construed as a potential conflict of interest.

## References

[B1] Alexander L. (2017). Production of triploid *Hydrangea macrophylla* via unreduced gamete breeding. HortScience 52, 221–224. 10.21273/HORTSCI11358-16

[B2] Borchert T. Gawenda I. (2010). Development and application of high-throughput amplified fragment length polymorphism technique in *Calluna vulgaris* (*Ericaceae*). Electr. J. Biotech. 13, 7–8. 10.2225/vol13-issue2-fulltext-3

[B3] Cerbah M. Mortreau E. Brown S. Siljak-Yakovlev S. Bertrand H. Lambert C. (2001). Genome size variation and species relationships in the genus *Hydrangea*. Theor. Appl. Genet. 103, 45–51. 10.1007/s001220000529

[B4] Chen H. Lu C. Jiang H. Peng J. (2015). Global transcriptome analysis reveals distinct aluminum-tolerance pathways in the Al-accumulating species *Hydrangea macrophylla* and marker identification. PLoS ONE 10:e0144927. 10.1371/journal.pone.0144927 26660093 PMC4682798

[B5] Cheng F. Wu J. Cai C. Fu L. Liang J. Borm T.. (2016). Genome resequencing and comparative variome analysis in a *Brassica rapa* and *Brassica oleracea* collection. Sci. Data 3:160119. 10.1038/sdata.2016.119 27996963 PMC5170593

[B6] Dolezel J. Greilhuber J. Suda J. (2007). Estimation of nuclear DNA content in plants using flow cytometry. Nat. Protoc. 2:2233. 10.1038/nprot.2007.310 17853881

[B7] Gruber K. (2017). Agrobiodiversity: the living library. Nature 544, 8–10. 10.1038/544S8a 28445449

[B8] Guérin V. (2002). Hydrangea: Acquisitions Nouvelles et Applications. Paris: INRA-quae.

[B9] Gürtler S. Klocke E. Schrader O. (2013). Mutagenese und Polyploidisierung zur Schaffung neuer genetischer Variabilität bei der Hortensie (*Hydrangea macrophylla*). J. Cultiv. Plants 65, 273–284. 10.5073/JfK.2013.07.03

[B10] Hokanson S. Szewc-McFadden A. Lamboy W. McFerson J. (1998). Microsatellite (SSR) markers reveal genetic identities, genetic diversity and relationships in a *Malus* × *domestica* Borkh. core subset collection. Theor. Appl. Genet. 97, 671–683. 10.1007/s001220050943

[B11] Jones K. D. Reed S. M. Rinehart T. A. (2007). Analysis of ploidy level and its effects on guard cell length, pollen diameter, and fertility in *Hydrangea macrophylla*. Hortscience 42, 483–488. Available online at: http://handle.nal.usda.gov/10113/17762

[B12] Lin T. Zhu G. Zhang J. Xu X. Yu Q. Zheng Z.. (2014). Genomic analyses provide insights into the history of tomato breeding. Nat. Genet. 46, 1220–1226. 10.1038/ng.3117 25305757

[B13] Lund B. Ortiz R. Skovgaard I. Waugh R. Andersen S. (2003). Analysis of potential duplicates in barley gene bank collections using re-sampling of microsatellite data. Theor. Appl. Genet. 106, 1129–1138. 10.1007/s00122-002-1130-y 12671763

[B14] McClintock B. (1929). A cytological and genetical study of triploid maize. Genetics 14, 180–222. 17246573 10.1093/genetics/14.2.180 PMC1201029

[B15] Meier F. (1990). Tellerhortensien-Züchtungen. Wädenswil: Eidgenössische Forschungsanstalt für Obst-, Wein- und Gartenbau.

[B16] Möhring H. K. Kuhlen H. Bosse G. (1956). Die Hortensien: ihre Geschichtliche Entwicklung, Systematik, Anatomie und Morphologie, Züchterische Bearbeitung, Sortenentwicklung und Kultur im Erwerbsgartenbau. Aachen: Verlag Deutsche Gärtnerbörse.

[B17] Reed S. M. Rinehart T. A. (2007). Simple sequence repeat marker analysis of genetic relationships within *Hydrangea macrophylla*. J. Am. Soc. Hort. Sci. 132, 341–351. Available online at: http://handle.nal.usda.gov/10113/7485

[B18] Rinehart T. A. Scheffler B. E. Reed S. M. (2006). Genetic diversity estimates for the genus *Hydrangea* and development of a molecular key based on SSR. J. Amer. Soc. Hort. Sci. 131, 787–797. Available online at: http://handle.nal.usda.gov/10113/21586

[B19] Sattler M. C. Carvalho C. R. Clarindo W. R. (2016). The polyploidy and its key role in plant breeding. Planta 243, 281–296. 10.1007/s00425-015-2450-x 26715561

[B20] Spellerberg B. (2017). Aktuelle Entwicklungen bei der Deutschen Genbank Zierpflanzen. Julius-Kühn-Archiv 457, 15–17. 10.5073/jka.2017.457.001

[B21] Uemachi T. Okumura A. (2012). The inheritance of inflorescence types in *Hydrangea macrophylla*. J. Jap. Soc. Hort. Sci. 81, 263–268. 10.2503/jjshs1.81.263

[B22] Van Huylenbroeck J. Leus L. van Bockstaele E. (2005). Interploidy crosses in roses: use of triploids. Acta Hort. 690, 109–112. 10.17660/ActaHortic.2005.690.15

[B23] Varshney R. K. Saxena R. K. Upadhyaya H. D. Khan A. W. Yu Y. Kim C.. (2017). Whole-genome resequencing of 292 pigeonpea accessions identifies genomic regions associated with domestication and agronomic traits. Nat. Genet. 49, 1082–1088. 10.1038/ng.3872 28530677

[B24] Wang X. Cheng Z.-M. Zhi S. Xu F. (2016). Breeding Triploid Plants: a Review. Czech, J. Genet. Plant Breed. 52, 41–54. 10.17221/151/2015-CJGPB

[B25] Zonneveld B. (2004). Genome size in *Hydrangea*, in Encyclopedia of Hydrangeas, eds van Gelderen J. van Gelderen D. M. (Portland, OR: Timber Press), 245–251.

